# The detect consensus report on Attention Deficit/Hyperactivity Disorder and its management among Turkish children and adolescents (Detect: consensus report on ADHD among Turkish youth)

**DOI:** 10.3389/fpsyt.2024.1372341

**Published:** 2024-03-20

**Authors:** Hakan Öğütlü, Özlem Meryem Kütük, Ali Evren Tufan, Aynur Pekcanlar Akay, Özlem Yildiz Gündoğdu, Eyüp Sabri Ercan

**Affiliations:** ^1^ Child and Adolescent Psychiatry Department, Cognitive Behavioral Psychotherapies Association, Ankara, Türkiye; ^2^ Child and Adolescent Psychiatry Department, Medical Faculty, Baskent University, Adana, Türkiye; ^3^ Child and Adolescent Psychiatry Department, Medical Faculty, Bolu Abant Izzet Baysal University, Bolu, Türkiye; ^4^ Child and Adolescent Psychiatry Department, Medical Faculty, Dokuz Eylul University, Izmir, Türkiye; ^5^ Child and Adolescent Psychiatry Department, Medical Faculty, Kocaeli University, Kocaeli, Türkiye; ^6^ Child and Adolescent Psychiatry Department, Medical Faculty, Ege University, Izmir, Türkiye

**Keywords:** ADHD, attention deficit, hyperactivity, consensus, report, detect, diagnosis, treatment

## Abstract

Attention Deficit/Hyperactivity Disorder (ADHD) is one of the most common and heritable neurodevelopmental disorders which may last through the life-span. A consensus report on diagnosis and management of ADHD among Turkish youth was prepared previously. However, the participants as well as the management options were rather limited and developments in the past decade necessitated a revision and update of the consensus. Therefore, this review aims to summarize the consensus among Child and Adolescent Psychiatrists from Türkiye on the nature and management of pediatric ADHD. For those aims, the etiology of ADHD, diagnostic and evaluation process, epidemiology, developmental presentations, differential diagnoses and comorbidities, course/outcome and pharmacological as well as non-pharmacological management options were reviewed and suggestions for clinical practice are presented. Since ADHD is a chronic disorder with wide-ranging effects on functionality that is frequently accompanied by other mental disorders, a multidimensional therapeutic approach is recommended. However, since the disorder has neurobiological basis, pharmacotherapy represents the mainstay of treatment. Additional therapies may include psychosocial therapy, behavioral therapy, school-based therapeutic approaches, and family education. This review provides recommendations for ADHD at the national and global levels. It contains information about ADHD that will contribute to and facilitate clinicians’ decision-making processes. It is advisable to consider this guideline in clinical practice.

## 1 Background

Attention Deficit/Hyperactivity disorder (ADHD) is one of the most common and heritable neurodevelopmental disorders which may last through the life-span (1). Currently neurobiological factors are known to play the most important roles in its etiology although environmental exposures may also affect symptom severity, comorbidities and prognosis (1). Reflecting the importance of neurobiological factors, the current standard of treatment is pharmacological (1). There are various national clinical guidelines for its assessment and management although most are from the Western countries (2–5). A consensus report on diagnosis and management of ADHD among Turkish youth was prepared previously (6). However, the participants as well as the management options were rather limited and developments in the past decade necessitated a revision and update of the consensus. Therefore, this review aims to summarize the consensus among Child and Adolescent Psychiatrists from Türkiye on the nature and management of pediatric ADHD. For those aims, the etiology of ADHD (in terms of genetics, neurobiology, neurophysiology, neuroimaging and neuropsychology), diagnostic and evaluation process, epidemiology, developmental presentations, differential diagnoses and comorbidities, course/outcome and pharmacological as well as non-pharmacological management options were reviewed and suggestions for clinical practice are presented.

## 2 Main text

‘DETECT’, an acronym derived from Turkish, stands for the consensus report on ADHD among Turkish youth, a designation that is notably appealing due to its coincidental English meaning. The detailed content is included in the following sections.

## 3 Etiology

Attention Deficit/Hyperactivity Disorder (ADHD) is a developmental disorder of neurobiological origin involving an interaction between many factors. The etiology of ADHD will be discussed under the headings of genetics, neurobiology, neurophysiology, neuroimaging, and neuropsychology.

### 3.1 Genetics

Genetic factors play a fundamental role in the development of ADHD. As suggested by twin studies, genetic factors may explain up to 70.0% to 80.0% of the variance in the diagnosis of ADHD. The risk of ADHD is 5 to 9-fold higher in siblings and parents of children with ADHD (1).

Molecular genetic studies have also strongly pointed out to an association between a diagnosis of ADHD and more than 100 genetic variations that particularly affect the functions of the dopaminergic and noradrenergic systems. These findings have been corroborated by candidate gene studies, in which the highest effect size was reported for Dopamine D4 receptor gene (DRD4) and Dopamin Transporter 1 (DAT1). Other genes include Dopamin D4 receptor gene (DRD5), SNAP 25 (Synaptosomal-Associated Protein 25kDa), and similar ones (7, 8).

Genome wide association studies (GWAS) have suggested that approximately 40.0% of the inheritance in ADHD may be associated with multiple and extensive genetic variations, with effect size ranging between low and moderate (9). While GWAS research does not support a role for single nucleotide polymorphisms in ADHD, the contribution of certain potential genes involved in the development of the nervous system has been underscored in these studies. The gene effect may be more prominent at the level of endophenotypes rather than syndromes, and research directed at endophenotypes may provide more valuable information regarding the detection of the disease risk (10).

The results of the published studies, the heterogeneity of the ADHD phenotypes, as well as the symptomatic variability observed through the lifespan suggest that the disorder results from the interaction between multiple genes, each with a low effect size, as well as interaction between genes and environment (11).

### 3.2 Neurobiology

Appropriate levels of dopamine (DA) and norepinephrine (NA) in the prefrontal cortex are required for executive functions (EF), including attention. Dopaminergic activity within the corticostriatal pathway is involved in motor functions and impulse control, while NA plays a role in the shift of attention in the parietal lobes, an in regulation of wakefulness in the brainstem. Since medications used for the treatment of this disorder have an effect on both of these cathecolamines, dopaminergic and noradrenergic systems are thought to play a fundamental role in the neurobiology of ADHD (12).

Glutamate is a DA regulator in the prefrontal cortex and striatum. A possible role for serotonergic and cholinergic systems in the development of ADHD has also been proposed, based on the facts that the former is involved in impulse control, while the latter is involved in memory and cognitive functions (13–15).

### 3.3 Neurophysiology

Studies on cranial electrical activity and blood flow have shown reduced blood flow and activity in the prefrontal cortex, parietal lobe, and basal ganglia of individuals diagnosed with ADHD (1, 16). Electroencephalography (EEG) studies have demonstrated increased theta wave activity and reduced alpha and beta wave activity in ADHD that were restored to normal with treatment (16, 17). Event-related potential studies showed smaller N2 and P3 amplitudes among ADHD patients, suggesting that this might be related to impulse control problems that may be alleviated with treatment (16–18). Also, ADHD may be related with problems in brain areas linked with daytime dreaming (19).

### 3.4 Neuroimaging

Until now, imaging studies in children diagnosed with ADHD showed reduced volume in the prefrontal cortex, basal ganglia, corpus callosum, and cerebellum (20). Some of these changes may improve with aging and treatment. Also, longitudinal studies suggested that children with ADHD may have a developmental delay in cortical maturation (21).

Studies using diffusion tensor imaging, which is a magnetic resonance imaging (MRI) method to assess the structure of the white matter, showed diffuse alterations in the structure of white matter in individuals with ADHD (22). Studies using functional MRI (fMRI) indicated reduced activity of the fronto-striatal, fronto-parietal, and fronto-cerebellar circuits involved in cognitive functions as well as increased activity in the default mode circuit. Although recent data suggest that children with ADHD may exhibit diffuse differences in cranial circuit activity, these differences may vary depending on the tasks and sample characteristics (23).

### 3.5 Neuropsychology

Several neuropsychological models have been proposed to explain the occurrence of ADHD including the “Executive Dysfunction”, “Cognitive Energy Model”, “Reward Sensitivity”, “Bekleme Direnci” or “Dual Pathway Model” (24). Based on the executive dysfunction model, the fundamental problem in ADHD is the inability to prevent the undesired responses (24). The Cognitive Energy Model posits a disordered control of the level of excitability and activity in ADHD. On the other hand, Reward Sensitivity and Delay Aversion Models are closely related. According to the former, responses are closely linked with reinforcement, with the ADHD patient having a desire to obtain quick rewards for responses and lacking patience for fulfillment of the reward. Tasks involving limited reinforcement are avoided.

Delay Aversion Model proposes that it is the duration of waiting rather than the presence/extent of reward and/or reinforcement that plays a role in the development of symptoms. According to this model, the internal restlessness and motor activity result from the inability to tolerate waiting. In the “Dual Pathway” model, the ADHD symptoms arise due to deficits associated with impulses (delay aversion and reward sensitivity) as well as with EF. Thus, both elements are required for the emergence of ADHD symptoms (24).

Published data regarding these models is far from being conclusive (25, 26). Neuropsychologic dysfunction may not be present in all children diagnosed with ADHD, and the problems identified through testing may have limited effect on daily life (27). Since the positive predictive value and specificity of neuropsychological test batteries are low, clinical assessments are important for establishing a diagnosis (28).

## 4 Diagnosis

### 4.1 Diagnostic Criteria

Diagnosis of ADHD is mainly based on the diagnostic criteria listed in the Diagnostic and Statistical Manual of Mental Disorders (DSM) and International Classification of Diseases (ICD). For a diagnosis of ADHD, symptoms of hyperactivity, impulsivity, and inattention inappropriate for chronological age/developmental level should be observed in more than one situation and be associated with functional impairment (1). Symptoms occur early in the life course. DSM-5 requires that at least some symptoms should have an onset prior to 12 years of age (28). Based on the predominant symptom category, the disorder may be classified as predominantly inattentive, predominantly hyperactive and impulsive, or combined ADHD (28).

### 4.2 Diagnosis and evaluation

Evaluation of ADHD requires a detailed clinical and developmental assessment in addition to comprehensive evaluation of history of complaints. It should be determined whether the symptoms may be better accounted for by other disorders (e.g. hearing impairment presenting as inattention), and more than one source of information should be utilized whenever possible. The diagnosis is based on clinical examination (28).

Although standard ADHD assessment tools such as the Disruptive Behaviour Rating Scale (29) are helpful in assessing ADHD or response to treatment, they cannot replace detailed history taking. Structured interviews may also be valuable in a clinical context.

### 4.3 Epidemiology

The reported global prevalence of ADHD is 5.3% (6.5% in children, and 2.7% in adolescents) (30). In community samples, the Predominantly Inattentive type may be the most frequent presentation (31). ADHD has been found to occur 2.5-3.0 and 6.0-9.0 times more frequently in males in community and clinical samples, respectively (31), with reduced male predominance with aging. The observed gender difference has been attributed to detection of hyperactivity and disruptive behaviors more readily among boys as well as to the symptomatic variation with age and development (1, 30). While the predominantly impulsive presentation is more common during early childhood, the combined presentation is more common in clinical samples consisting of school children (1, 30). On the other hand, the predominantly inattentive presentation may be more common among girls (1, 31).

One of the first studies from Türkiye on the prevalence and course of ADHD reported a prevalence rate of 12.7%, among students in the first four year of primary school (32). In a more recent, multi-center study, the prevalence of ADHD associated with functional impairment was found to be 12.4% (33).

### 4.4 Developmental presentation

Some cases with ADHD may present with in infancy with difficult temperament. In pre-school children, the presenting symptoms may include bouts of anger, sleep disturbances, injuries and accidents, and delayed or impaired speech (30, 34). Again, inability to complete tasks, hyperactivity, peer problems, problems associated with initiation of sleep, and opposition may be the predominant symptoms during the pre-school period (30–36). On the other hand, presenting symptoms during the school age may include academic problems, excessive talking/butting into conversation, inability to follow instructions, organisation problems, peer problems, and inability to sit still in the classroom (36).

ADHD symptoms may continue during the adolescents and may aggravate the impulsive behaviors that are part of the natural development during adolescence. As patients age, hyperactivity may be replaced by anxiety, while inattention and disorganized behaviors continue (30–36) A diagnosis of ADHD during adolescence may also be associated with anger control problems as well as substance and alcohol abuse (30–37).

### 4.5 Differential diagnosis

ADHD may be confused with many other medical disorders. Hearing and vision impairment, genetic disorders, absence type epilepsy, thyroid disorders, sleep disorders, lead poisoning, iron deficiency anemia, vitamin B12 and/or folate deficiency, recent infections, and drug side effects may mimic ADHD (2, 6). Also, some other psychiatric disorders should be considered in the differential diagnosis, including major depressive disorder, bipolar disorder, anxiety disorders, specific learning disorder, tic disorders, autism-spectrum disorders, behavioral disorders, oppositional defiant disorder, and substance abuse disorder (2–6, 36, 37).

### 4.6 Comorbid conditions

Comorbid conditions are common among children with ADHD (1–6, 36, 37). The most common comorbid conditions include the Disruptive Behavior Disorders (30.0% to 80.0%), Learning Disorders (15.0% to 40.0%), Anxiety Disorder (25.0% to 35.0%), and Major Depressive Disorder (15.0% to 20.0%) (1–6, 36–38). Also, ADHD may be accompanied by bipolar disorder, tic disorder, substance use disorders, specific speech disorders (particularly in girls), developmental coordination disorder, and sleep disorder (1–6, 36–38). Symptoms of hyperactivity and impulsivity may increase the risk of behavioral problems, while concomitant diagnoses of inattention and learning disorder may reflect the shared genetic risk factors (1–6, 36–38). In one study from our country, 41.3% of ADHD cases had one comorbid condition, while 12.0% and 30.0% had at least two and three concomitant disorders, respectively, the most common being disruptive behavior, major depressive disorder, and anxiety disorder (38).

Concomitant disorders in patients with ADHD may vary according to developmental stage (36–38). While the most common concomitant disorders in preschool period include reactive attachment disorder, autism spectrum disorders, and developmental delays, those during the school age include anxiety disorder, disruptive behavior disorder, learning disorder, and tic disorder. In adolescents, more common concomitant disorders are alcohol/substance abuse problems, internet addiction, and bipolar disorder (1–6, 36–39).

### 4.7 Course and outcome

The diagnosis of ADHD persists into adolescence and adulthood in 60 to 85% and 40 to 60% of the cases, respectively (1–6, 36–39). In a review examining the clinical practices in several areas including Hong Kong, Singapore, South Korea, Türkiye, and Africa, it has been found that patients may fail to adhere to therapy during transition from childhood to adolescence (40). Risk factors for persistence of ADHD into adulthood include learning disorders, symptom severity, number of concomitant disorders, presence of a family history of mental disorders and ADHD, low socioeconomic level, and delayed/inadequate management (41).

### 4.8 Treatment

In this section, ADHD treatment will be covered under the headings of pharmacological, psychosocial, and alternative treatments. Since ADHD is a chronic disorder with wide-ranging effects on functionality that is frequently accompanied by other mental disorders, a multidimensional therapeutic approach is recommended (1–6). However, since the disorder has neurobiological basis, pharmacotherapy represents the mainstay of treatment (1–6, 42) Additional therapies may include psychosocial therapy, behavioral therapy, school-based therapeutic approaches, and family education (43).

#### 4.8.1 Pharmacological treatments

Current pharmacological treatments in ADHD include stimulant and non-stimulant agents. Due to the chronic nature of ADHD, treatment interruptions are not recommended unless absolutely required (44).

##### 4.8.1.1 Psychostimulant agents

These represent the most preferred and most commonly used drugs. Agents in this category include different formulations of methylphenidate (MPH), mixed amphetamine salts, dextroamphetamine, and lysdexamphetamine dimesylate (44, 45). Psychostimulant agents prevent the reuptake of dopamine and noradrenaline into presynaptic neurons, in addition to increasing dopamine release from presynaptic neurons. Generally, they are believed to increase the catecholamine levels in the striatum and to regulate the prefrontal cortical functions, via the frontostriatal pathway (44, 45).

In our country, three different formulations containing MPH are available (short-acting Ritalin™ 10 mg tablets, and long-acting Concerta™ 18, 27, 36, 54 mg capsules, and Medikinet Retard™ 10, 20, 30 and 40 mg tablets). The short-acting form is generally prescribed in divided doses (two-three times/daily), at a daily dose of 0.3 to 1.5 mg/kg/day. The suitable dose range for children and adolescents is between 10 and 60 mg/day. The long-acting formulations are prescribed at a daily dose of 0.4-1.8 mg/kg/day, with a dose interval of 18-72 mg/day (44).

As it is not bound to plasma proteins, MPH is rapidly metabolized and 90.0% of the administered dose is excreted in the urine. Amphetamine containing preparations utilized clinically such as dextroamphetamine sulfate (Dexedrine™), amphetamine/dextroamphetamine salts (Adderal™), and lysdexamphetamine (Vyvanse™) are currently not available in Türkiye. The reported effect size on ADHD symptoms for methylphenidate in studies involving school children, adolescents, and adults is 0.9 (0.99 for short acting formulations [95% Confidence Interval=0.88-1.10], 0.95 for long acting formulations [95% Confidence Interval = 0.85-1.10]) (44, 45).

Psychostimulants are associated with improvements not only in the main symptoms of ADHD, but also in academic performance and social relationships (45). Side effects of mild-to-moderate severity are reported in 4% to 10% of the patients. The most frequently reported side effects include sleeplessness, loss of appetite, abdominal pain, gastrointestinal problems, headache, dizziness, and palpitations. More rarely anxiety, irritability, emotional lability, and bursts of anger may be seen. Most of these side effects tend to decline after the first week of treatment, and may be resolved with dose reduction or discontinuation (44, 45). Tic disorders are commonly seen in children or adolescents with ADHD independent of the treatment. Therefore, the role of stimulant agents alone in triggering tics is unclear. Current weight of opinion suggests that although such treatments may induce or aggravate tics in children with underlying genetic predisposition, this alone does not constitute a contraindication for their use (44–46). Use of stimulant agents is not associated with an increased risk of sudden death or cardiovascular complications. Therefore, cardiac examination and electrocardiographic assessments are not required in patients without underlying heart disease or familial risk factors (44, 45). Treatment with methylphenidate does not affect the final weight and height achieved in adulthood. Also, these agents have been shown to lack the potential for addiction, and conversely, may reduce the risk of alocohol, substance, or nicotine use disorder by providing early treatment for ADHD symptoms (44, 45).

#### 4.8.2 Non-stimulating agents

##### 4.8.2.1 Atomoxetine

Atomoxetine is the first non-stimulant agent approved by the FDA for the treatment of children with ADHD over 6 years of age. It is a selective and effective presynaptic noradrenalin transporter inhibitor, and is also believed to have very weak affinity for serotonin/dopamine transporters (42, 44). It is not associated with a potential for addiction, as it does not affect DA levels in the striatum and nucleus accumbens (42). It has been reported that it may be preferable over stimulating agents in ADHD patients with comorbid substance abuse, anxiety, or tic disorders, or for symptomatic control in early morning or late night hours (2–6, 44, 47–49).

In Türkite, Setinox™ and Strattera™ are the examples of brand names. ATX is administered at daily doses of 0.5-1.8 mg/kg, with one or two daily doses depending on tolerability. The onset of effect is observed at the end of first treatment week, while in some cases there may be a delay of up to 1 month. Single or twice daily administration is not associated with differential efficacy (2–6, 44, 47, 49). The treatment has been reported to be effective on symptoms of both inattention, hyperactivity, and impulsivity, with an effect size of 0.62 (44). The most common side effects include lack of appetite, dry mouth, nausea, sweating, sleeplessness, palpitations, increased blood pressure, and sedation. Most of these side effects resolve with continued treatment. As compared to stimulant agents, ATX has been reported affect the duration of sleep and rhythm to a lesser extent (44).

##### 4.8.2.2 Alpha 2- Adrenergic agonists

Alpha-2 adrenergic agonists (clonidine and guanfacine) act by stimulating presynaptic alpha-2 receptors in the central nervous system. Their effect on ADHD symptoms is thought to occur via an increase in NA levels in the prefrontal cortex and an increase in noradrenergic tonus in locus ceruleus (2–6, 42, 44, 48, 49). Controlled-release formulations of both agents have been approved by FDA for monotherapy or in combination with other drugs for ADHD patients aged over 6 years. They may be used alone or in association with other medicines in patients with opposing behavior, aggression, or tic disorder, or in subjects who cannot benefit or tolerate stimulant agents (2–6, 42, 44). Relatively frequent but temporary side effects include drowsiness, headache, fatigue, and sedation. Effects on hypotension and pulse may last longer, with clonidine potentially being more risky in this regard. For these agents, gradual dose increments and tapering off are recommended (2–6, 42, 44, 46–49).

##### 4.8.2.3 Antidepressants

Imipramine and desypramine, which are tricyclic antidepressants, are not preferred in the treatment of ADHD due to their cardiac side effects. They may be used alone or in combination in patients with comorbid anxiety, depression, tic disorder, or nocturnal enuresis in case of resistance to other treatments. As with alpha-2 agonists, the treatment should be initiated and tapered using gradual dose adjustments, an electrographic assessment should be performed prior to therapy and dose increments, and the dosage should not exceed 2.5 mg/kg daily (2–6, 42, 44).

Another antidepressant agent, bupropion, prevents noradrenaline and dopamine reuptake. It is similar to psychostimulant agents in terms of chemical structure, and it is more commonly used for smoking cessation. In a recent review, although bupropion was found to be useful for symptom control in children and adolescents with ADHD, a recommendation to consider this agent only in resistant cases was made due to its side effects such as epileptic seizures (50).

Despite several studies with other antidepressants such as reboxetine or venlafaxine, their routine clinic use is currently not recommended (42, 44, 50).

### 4.9 Psychosocial treatments

Approximately 20 to 30% of patients with ADHD do not respond adequately to pharmacotherapy. In most of these cases, psychosocial treatments may offer some help. Until now, a treatment benefit was shown for psychosocial therapies such as cognitive therapy/parent education programs, social skills training, mindfulness training, family therapy, and school-based interventions (43, 44).

### 4.10 Alternative treatments

Some studies have evaluated alternative treatments such as omega 3 and 6 fatty acids, dietary interventions, zinc supplementation, and neurobiofeedback. However, recent reviews suggest that such interventions lack efficiency in the treatment of ADHD (51–53).

## 5 Conclusions

This review provides recommendations for ADHD at the national and global levels. Diagnosing ADHD is a process that must be carefully pursued. A multidimensional therapeutic approach is recommended in the treatment of ADHD. The review contains information about ADHD that will contribute to and facilitate clinicians’ decision-making processes. It is advisable to consider this guideline in clinical practice. This guide and similar other guides need to be renewed over time and as treatment needs to change.

## Author contributions

HÖ: Data curation, Formal Analysis, Writing – original draft, Writing – review & editing. ÖK: Writing – original draft, Writing – review & editing. AT: Writing – original draft, Writing – review & editing. AA: Writing – review & editing. ÖG: Writing – review & editing. EE: Writing – review & editing.
